# Recent preclinical and clinical advances in radioimmunotherapy for non-Hodgkin’s lymphoma

**DOI:** 10.37349/etat.2024.00213

**Published:** 2024-02-28

**Authors:** Hiroki Goto, Yoshioki Shiraishi, Seiji Okada

**Affiliations:** Institute of Oncology Research, Switzerland; ^1^Division of Radioisotope and Tumor Pathobiology, Institute of Resource Development and Analysis, Kumamoto University, Kumamoto 860-0811, Japan; ^2^Division of Hematopoiesis, Joint Research Center for Human Retrovirus Infection, Kumamoto University, Kumamoto 860-0811, Japan; ^3^Radioisotope Center, Institute of Resource Development and Analysis, Kumamoto University, Kumamoto 860-0811, Japan

**Keywords:** Radioimmunotherapy, monoclonal antibody, non-Hodgkin’s lymphoma, pre-targeted radioimmunotherapy, immuno-positron emission tomography

## Abstract

Radioimmunotherapy (RIT) is a therapy that combines a radioactive nucleotide with a monoclonal antibody (mAb). RIT enhances the therapeutic effect of mAb and reduces toxicity compared with conventional treatment. The purpose of this review is to summarize the current progress of RIT for treating non-Hodgkin’s lymphoma (NHL) based on recent preclinical and clinical studies. The efficacy of RIT targeting the B-lymphocyte antigen cluster of differentiation 20 (CD20) has been demonstrated in clinical trials. Two radioimmunoconjugates targeting CD20, yttrium-90 (^90^Y)-ibritumomab-tiuxetan (Zevalin) and iodine-131 (^131^I)-tositumomab (Bexxar), have been approved in the USA Food and Drug Administration (FDA) for treating relapsed/refractory indolent or transformed NHL in 2002 and 2003, respectively. Although these two radioimmunoconjugates are effective and least toxic, they have not achieved popularity due to increasing access to novel therapies and the complexity of their delivery process. RIT is constantly evolving with the identification of novel targets and novel therapeutic strategies using newer radionuclides such as alpha-particle isotopes. Alpha-particles show very short path lengths and high linear energy transfer. These characteristics provide increased tumor cell-killing activities and reduced non-specific bystander responses on normal tissue. This review also discusses reviewed pre-targeted RIT (PRIT) and immuno-positron emission tomography (PET). PRIT potentially increases the dose of radionuclide delivered to tumors while toxicities to normal tissues are limited. Immuno-PET is a molecular imaging tracer that combines the high sensitivity of PET with the specific targeting capability of mAb. Immuno-PET strategies targeting CD20 and other antigens are currently being developed. The theragnostic approach by immuno-PET will be useful in monitoring the treatment response.

## Introduction

Non-Hodgkin’s lymphoma (NHL) is the most frequent hematological malignancy in adults. NHL is classified into two groups in clinical practice: the indolent (low-grade) lymphoma and the aggressive (high-grade) lymphoma. Follicular lymphoma (FL) is the most frequent indolent NHL, and diffuse large B-cell lymphoma (DLBCL) represents the most frequent type of aggressive NHL. Antibody-based therapies have been developed in the treatment of NHL [1–3]. The administration of rituximab, a monoclonal antibody (mAb) targeting cluster of differentiation 20 (CD20), has markedly improved the treatment response and disease outcome of NHL [1]. However, indolent NHL is not curable and a certain number of patients with an indolent NHL (about 40% of FL patients) eventually experience relapsed or refractory disease with a more aggressive histology [4]. The aggressive types of NHL are heterogenous and some of them still relapse after chemotherapy (at least one-third of DLBCL patients are refractory to first-line chemotherapy) [5, 6]. Thus, there is a need for alternative strategies that are less toxic and more effective for patients with relapsed or refractory NHL compared with conventional therapy.

Radioimmunotherapy (RIT) is a therapy to selectively deliver therapeutic radionuclides to tumor lesions by conjugating radionuclides to mAbs while normal tissue toxicity is limited. The effectiveness and safety of RIT such as Zevalin [yttrium-90 (^90^Y)-ibritumomab-tiuxetan] and Bexxar [iodine-131 (^131^I)-tositumomab] have been proven relative to rituximab in relapsed or refractory NHL. New, effective therapies have been developed and are playing against the spread of RIT in the NHL. Indeed, the sales of Bexxar were discontinued in 2014 for commercial reasons. Zevalin is still available in the market but its sales have reportedly declined compared with a decade ago. However, most of the relapsed or refractory NHL patients are elderly and their therapeutic options are limited. Thus, RIT will be suitable for patients suffering from NHL.

To prevent long-time retention of the radionucleotides in the blood and decrease toxicity, the concept of pre-targeted RIT (PRIT) has been proposed [7]. In PRIT, a non-radioactive antibody is administered and allowed to bind to a tumor antigen. Subsequently, a radioactive payload is injected and captured by a cell-bound antibody. PRIT is currently being evaluated in both preclinical and clinical investigations.

Positron emission tomography (PET) using radiolabeled mAb (immuno-PET) is a new imaging modality that combines mAb and PET radionucleotides and provides pharmacokinetic and pharmacodynamic information. PET using fluorine-18 (^18^F)-fluorodeoxyglucose (FDG) has been recommended for relapse detection and therapy assessment in NHL. Nevertheless, ^18^F-FDG is not tumor-specific and does not allow the selection of patients for targeted therapy since FDG evaluates only glucose metabolism. To develop immuno-PET using mAb, a positron emitter that shows a long half-life enough for blood clearance of antibodies would be ideal. Recent reports have described long-lived PET radionuclides such as zirconium-89 (^89^Zr) are trapped inside the tumor cell after antibody internalization and thus produce high-resolution, excellent contrast imaging. With current advancements, immuno-PET could be used in the evaluation of NHL as a novel imaging for the selection of patients and treatment response assessment.

This review summarizes the recent preclinical and clinical advances in RIT for treating NHL and discuss future applications of RIT and radiotheragnostic agents.

## β-Particle RIT

In RIT, either β- or α-emitting radionucleotide can be linked to the antibodies for delivery of radiation to the tumor cells. Therapeutic radionucleotides are selected on the basis of their particle emission, half-life, energy, and path length. The path length is defined by the distance that the charged particles can travel. The treatable tumor size is determined by path length. Potential β- and α-emitting radionucleotides for RIT of NHL are shown in Table 1.

**Table 1:** β-Emitting and α-emitting radionucleotides for RIT of NHL

Radionucleotides	Emission	Half-life	Energy (keV)	Path length
β-Emitting radionucleotide				
^90^Y	β^–^	2.67 days	2,280	12 mm
^131^I	β^–^, γ	8.02 days	606	0.2–1 mm
Lutetium-177 (^177^Lu)	β^–^, γ	6.68 days	498	0.23 mm
α-Emitting radionucleotide				
Astatine-211 (^211^At)	α, electron capture (EC)	7.21 h	5,870 (41.8%), 7,450 (58.2%)	-
Plumbum-212 (^212^Pb)	α, β^–^, γ	10.64 h	6,051 (36%), 8,875 (64%)	-
Bismuth-213 (^213^Bi)	α, β^–^, γ	46 min	8,376	-
Actinium-225 (^225^Ac)	α, β^–^, γ	10 days	5,830, 6,341, 7,067, 8,376	-
Thorium-227 (^227^Th)	α, β^–^, γ	18.72 days	5,716, 6,038, 6,623, 6,819, 7,386	-

The path length of α-emitting radionucleotides is around 50–100 µm. Energies from α-emitting radionucleotides include those of daughter nucleotides. The data in parentheses indicates emission rates. -: no data

β-Particle radiation can exert a direct toxic effect on the cell bound by the antibody and eliminate surrounding tumor cells via cross-fire effect. The cross-fire effect can kill cells that have low levels of antigen expression and are not accessible to the antibody [8]. The therapeutic effect of CD20 mAb therapy can be enhanced by the antibody-conjugated β-emitting radionuclide [9]. β-Particle RITs targeting CD20 kill not only CD20^+^ lymphoma cells but also CD20^+^ normal B-cell. However, normal B-cells eventually recover because CD20 is not expressed on B-cell precursors and hematopoietic stem cells. Two β-particle RITs targeting CD20 have been approved for relapsed or refractory NHL. A summary of β-particle RITs evaluated in clinical trials of NHL is shown in Table 2.

**Table 2:** Summary of β-particle radioimmunotherapies evaluated in clinical trials of NHL

Radionucleotide-labeled antibody	Target	USA Food and Drug Administration (FDA) approval	Reference
^90^Y-ibritumomab-tiuxetan	CD20	Yes (February 2002)	[10–20]
^131^I-tositumomab	CD20	Yes (June 2003)	[21–24]
^90^Y-epratuzumab tetraxetan	CD22	No (Only phase I/II study)	[25]
^177^Lu-lilotomab satetraxetan	CD37	No (Only phase I/II study)	[26–30]

The first RIT is ^90^Y-labeled ibritumomab tiuxetan. In February 2002, ^90^Y-ibritumomab tiuxetan (Zevalin) was approved by the USA FDA to treat relapsed or refractory low-grade B-cell NHL. Ibritumomab is a murine variant of rituximab that targets the same CD20 epitope as rituximab. Tiuxetan is covalently bound to ibritumomab and chelates with indium-111 (^111^In, for imaging) or ^90^Y (for therapy). The patients need to receive non-radiolabeled rituximab to block normal B-cells in the blood circulation and in the spleen before ^90^Y-ibritumomab tiuxetan treatment. In a phase I/II study, ^90^Y-ibritumomab tiuxetan resulted in durable responses in patients with NHL including FL, DLBCL, non-follicular low-grade, and mantle cell lymphoma overall response rate (ORR) was 73%. Complete response (CR)/CR unconfirmed (CRu) was 51% and partial response (PR) was 22% [10]. The phase III randomized trial compared a single intravenous dose of ^90^Y-ibritumomab-tiuxetan with four doses of rituximab in 143 patients with relapsed or refractory low-grade, follicular, or transformed CD20^+^ transformed NHL [11]. The ^90^Y-ibritumomab-tiuxetan group showed a statistically significant higher ORR (80% *vs.* 56%) in comparison to the rituximab alone group. In patients with rituximab-refractory FL (no objective response to rituximab or time to progression of ≤ 6 months), ^90^Y-ibritumomab tiuxetan exhibited excellent effectiveness with 74% ORR (15% CR and 59% PR) [12]. The efficacy of ^90^Y-ibritumomab tiuxetan tended to be lower in the patients who relapsed after treatment with rituximab [13, 14]. In the phase II clinical study of ^90^Y-ibritumomab-tiuxetan as first-line monotherapy for FL, a single injection of ^90^Y-ibritumomab-tiuxetan achieved high response rates (56% CR/CRu and 31% PR) and was well tolerated [15]. Two doses of ^90^Y-ibritumomab-tiuxetan as initial therapy of advanced-stage FL showed excellent response rates (initial ORR was 94.4% and CR/CRu was 58.3%) in a phase II trial [16]. RIT consolidation with ^90^Y-ibritumomab-tiuxetan was highly effective for advanced-stage FL [17, 18] or DLBCL [19]. In addition, myeloablative conditioning with Zevalin (^90^Y-ibritumomab tiuxetan) plus 1,3-bis(2-chloroethyl)-1-nitrosourea (BCNU), etoposide, cytarabine, melphalan (BEAM, Z-BEAM) showed improved overall survival (OS) at 4 years with lower toxicity compared with total body irradiation-based conditioning regimens (81.0% *vs.* 52.7%) [20].

The second is ^131^I-labeled tositumomab. Tositumomab is a murine mAb targeting CD20. In June 2003, ^131^I-tositumomab (Bexxar) was approved to treat relapsed or refractory low-grade B-cell NHL by the USA FDA. Non-radiolabeled tositumomab is administered intravenously to bind normal B-cells in blood circulation and the spleen before the injection of ^131^I-tositumomab. In an integrated analysis of the five clinical studies, a single course of ^131^I-tositumomab administration showed high response rates in patients with relapsed/refractory low-grade or transformed low-grade NHL [21]. Response rates ranged from 47% to 68%, CR rates ranged from 20% to 38%, and the 5-year progression free survival (PFS) was 17% [21]. In treatment-naive advanced-stage FL, a single one-week course of ^131^I-tositumomab therapy was assessed as an initial treatment [22]. After ^131^I-tositumomab therapy, 95% had any response and 75% had a CR. Hematological toxicity was moderate without any transfusions. In a phase III randomized study, cyclophosphamide, hydroxydaunorubicin (adriamycin), oncovin (vincristine), and prednisone (CHOP) followed by ^131^I-tositumomab demonstrated significantly better 10-year PFS compared with rituximab-CHOP as an initial treatment for previously untreated patients with FL (56% *vs.* 42%) [23]. Autologous stem cell transplantation following high-dose RIT using ^131^I-tositumomab demonstrated improved OS and PFS in relapsed FL patients compared with conventional high-dose therapy [24]. Given these favorable outcomes, β-particle RIT remains the promising approach for patients with NHL although the marketing of Bexxar was discontinued in February 2014 due to a decline in usage.

CD22 is detected in more than 90% of patients with NHL including DLBCL and FL. Epratuzumab is a humanized mAb to CD22. In a phase I/II study, fractionated anti-CD22 RIT using ^90^Y-epratuzumab tetraxetan showed a high rate of durable CR and ORR in 41% to 73% of the patients with relapsed/refractory NHL [25]. High rates of CR/CRu (92%) and increased median PFS (24.6 months) were shown in patients with relapsed/refractory FL receiving the highest ^90^Y dose levels (> 30 mCi/m^2^).

CD37-targeting RIT has also been developed because CD37 is detected on not only mature normal B-cells but also the majority of B-cell NHL. Betalutin, ^177^Lu-lilotomab satetraxetan, consists of the β-particle ^177^Lu chelated to the chemical linker satetraxetan conjugated to the murine anti-CD37 mAb lilotomab. The bone marrow toxicity of ^177^Lu is relatively low due to its short β range (Table 1). In addition, γ rays emitted by ^177^Lu provide imaging of biodistribution and dosimetry measurements. The safety of ^177^Lu-lilotomab satetraxetan has been reported in relapsed CD37^+^ indolent NHL [26–30]. Treatment with ^177^Lu-lilotomab satetraxetan shows excellent therapeutic efficacy in NHL preclinical models [31–34]. ^177^Lu is also radiolabeled to ofatumumab which is a fully human anti-CD20 mAb and shows more efficient binding to CD20 antigen compared with rituximab. The therapeutic effect of ^177^Lu-ofatumumab was tested using the disseminated Raji lymphoma model [35]. When therapy was initiated 4 days after cell injection, 8.51 MBq of ^177^Lu-ofatumumab caused the elimination of bioluminescence-detectable tumors and no apparent effect on the whole-body.

The decreased level of CD20 expression is considered to be one of the major contributing factors for anti-CD20 mAb response [36]. Although the loss of CD20 antigen is assumed in the resistant mechanism of CD20-targeted therapy [37, 38], it is difficult to evaluate the frequency of CD20 loss because re-biopsy is not performed in most patients after CD20-targeted therapy. Thus, there is little information about the resistance mechanism of RIT targeting CD20.

In RIT targeting CD20, non-radiolabeled anti-CD20 mAb (rituximab) is injected prior to anti-CD20 radioimmunoconjugate (^90^Y-labeled ibritumomab tiuxetan and ^131^I-labeled tositumomab). Several lines of preclinical and clinical studies have shown administering the non-radiolabeled anti-CD20 mAb improves tumor targeting and prolongs the blood residence time of the radioimmunoconjugates. In previous studies using preclinical mouse models, circulating non-radiolabeled antibodies have the possibility to compete with the radioimmunoconjugate compromising the tumor uptake and therapeutic efficacy of the radioimmunoconjugate [39, 40]. To improve the response of RIT, the efficacy of dual-targeted RIT has been tested. Mattes et al. [41] evaluated the combination of an unconjugated humanized anti-CD20 mAb (veltuzumab) with a ^90^Y-epratuzumab tetraxetan in nude mice bearing Burkitt lymphoma (Ramos). In this investigation, tumor response and survival were improved using ^90^Y-epratuzumab tetraxetan along with non-radiolabeled veltuzumab compared with either of them alone. Weber et al. [42] tested combining rituximab with ^177^Lu-conjugated humanized anti-CD22 mAb, huRFB4, in a subcutaneous Raji lymphoma model. Treatment with ^177^Lu-conjugated huRFB4 significantly attenuated lymphoma growth and prolonged survival in comparison with ^177^Lu-conjugated rituximab. In a phase I study including 18 patients with relapsed aggressive B-cell NHL, the combination of two injections of ^90^Y-epratuzumab tetraxetan (222–555 MBq/m^2^) with four injections of veltuzumab was evaluated [43]. For ^90^Y-epratuzumab tetraxetan, the maximum tolerable dose was 222 MBq/m^2^ because of myelosuppression. Of 17 assessable patients, ORR was 53% including three (18%) CR and six (35%) PR. The combination therapy with ^90^Y-epratuzumab tetraxetan and veltuzumab was tolerable and promising in the elderly or patients who relapse or are not suitable for stem-cell transplantation.

## α-Particle RIT

RIT using β-particles has been already approved in patients with NHL, but its clinical application is gradually declining due to some limitations. β-Particles damage surrounding healthy tissues and provide the suboptimal killing of tumor cells because of relatively long tissue penetration ranges and low decay energies. α-Particles, in contrast, have a very short tissue penetration range (50–100 μm) and high linear energy transfer (Table 1); therefore, mAbs labeled with α-particles show high specific killing effects on tumor cells and minimal damage to surrounding normal tissue. α-Particle RIT is an attractive therapy for patients with NHL. A summary of preclinical and clinical investigations in α-particle RIT of NHL is shown in Table 3 and Table 4. Treatment with ^227^Th-1,4,7,10-tetraazacyclododecane-1,4,7,10-tetraacetic acid (DOTA)-p-benzyl-rituximab suppressed the lymphoma growth in nude mice bearing Burkitt lymphoma (Raji) and caused significantly longer survival than β-emitting ^90^Y-ibritumomab-tiuxetan [44]. ^212^Pb-rituximab significantly prolonged survival compared with rituximab and the ^212^Pb-isotypic control in a murine syngeneic lymphoma model [45]. Treatment with ^212^Pb-1,4,7,10-tetrakis(carbamoylmethyl)-1,4,7,10-tetraazacyclododecane (TCMC)-NNV003 (anti-CD37) demonstrated efficacy and safety in preclinical models of CD37-expressing chronic lymphocytic leukemia and NHL [46]. Treatment with ^213^Bi-rituximab was more effective in severe combined immunodeficient (SCID) mice bearing Burkitt lymphoma (Raji) than ^213^Bi or ^213^Bi anti-human epidermal growth factor receptor 2 (HER2)/neu [47]. Green et al. [48] assessed the anti-CD20 (1F5-B10) mAb labeled with the α-emitting radio-halogen ^211^At in both subcutaneous and disseminated lymphoma xenograft models. In a subcutaneous lymphoma xenograft model, high doses of ^211^At 1F5-B10 (48 µCi) treatment showed modest attenuation in lymphoma proliferation and slightly longer survival compared with no treatment. In a disseminated lymphoma model, a 15 µCi dose caused complete eradication of the lymphoma in 70% of mice. These results suggest α-particle RIT is more effective for small lymphoma cell clusters in a disseminated model because of its short ranges as expected. In mice bearing subcutaneous Raji lymphomas, ^225^Ac-labeled anti-CD20 ofatumumab treatment specifically killed lymphoma cells and showed dose-dependent curative therapeutic efficacy [49]. In a first-in-human dose-escalation phase I study, α-particle emitting ^227^Th-labeled anti-CD22 antibody (BAY 1862864) showed clinical safety and tolerability in patients with CD22-positive relapsed/refractory B-cell NHL [50].

**Table 3:** Summary of preclinical investigations in α-particle RIT of NHL

Radionucleotide-labeled antibody	Lymphoma	Comparison with ^90^Y-tiuxetan-ibritumomab	Reference
^227^Th-DOTA-p-benzyl-rituximab	Burkitt lymphoma (Raji)	Yes	[44]
^212^Pb-TCMC-rituximab	Mouse lymphoma (EL4-hCD20-Luc)	No	[45]
^212^Pb-TCMC-NNV003 (anti-CD37)	Burkitt lymphoma (Daudi), chronic lymphocytic leukemia (MEC-2)	No	[46]
^213^Bi-(*R*)-2-amino-3-(4-isothiocyanatophenyl)propyl)-*trans*-(*S*,*S*)-cyclohexane-1,2-diamine-pentaacetic acid (SCN-CHX-A”-DTPA)-rituximab	Burkitt lymphoma (Raji)	Yes	[47]
^211^At-1F5-B10 (anti-CD20)	Burkitt lymphoma (Ramos), mantle cell lymphoma (Granta-519)	No	[48]
^225^Ac-DOTA-ofatumumab	Burkitt lymphoma (Raji)	No	[49]

**Table 4:** Summary of clinical investigations in α-particle RIT of NHL

Radionucleotide-labeled antibody	Main finding	Reference
^227^Th-3,2-3,2-hydroxypyridinone (HOPO)-epratuzumab (anti-CD22, BAY 1862864)	BAY 1862864 (up to 6.1 MBq) showed safety and tolerability in 21 relapsed/refractory low- and high-grade NHL patients in a first-in-human dose-escalation phase I study.	[50]

There are several advantages in terms of increased cytotoxic effects on tumor cells and reduced non-specific bystander responses on normal tissue in α-particle RIT. However, α-particle RIT would be a challenge in the clinical setting because of the short half-life and low production capability. Further advancements in the method of production will reduce production costs and increase the use of α-particle RIT.

In contrast to β-particle radiation, α-particle radiation doesn’t target surrounding tumor cells and cells showing low levels of antigen expression due to its very short tissue penetration range. To target surrounding tumor cells and tumor heterogeneity, enhancing anti-tumor activities via bystander killing and immunogenic cell death needs to be explored in α-particle RIT [51, 52].

In α-particle RIT, low molecular weight ligands instead of antibodies are desirable for targeting moieties because they migrate into the cell nucleus more easily than antibodies and reduce toxicity. For the therapy of metastatic, castration-resistant prostate cancer, ^225^Ac-prostate-specific membrane antigen (PSMA)-617 shows a remarkable therapeutic efficacy against tumor cells [53, 54]. Similar strategies need to be developed in α-particle RIT for NHL.

α-Emitting radionucleotides can be used in combination with diagnostic imaging and therapeutic RIT. Most α-emitting radionucleotides emit photons during their decay. While the biodistribution of α-emitters such as ^211^At can be evaluated by single photon emission computed tomography (SPECT) imaging [55], obtaining high-quality SPECT imaging is difficult due to the low intensity of photons. The diagnostic imaging surrogates need to be developed to perform the assessment of pre-therapy in α-particle RIT.

## Advantages and disadvantages of RITs compared with antibody-drug conjugates

Theragnostics integrates diagnostic nuclear medicine and RIT, helping to predict the response and toxicity of RIT. The clinicians can manipulate RIT dosage according to the imaging data. In this setting, RIT must be performed in the hospital with the control area for radiation. Therefore, it is difficult to perform RIT in the general hospital. Antibody-drug conjugates (ADCs) are immunoconjugates composed of a mAb tethered to a cytotoxic drug (instead of a radionucleotide in RIT) via a chemical linker [56]. ADCs have bystander effects which are similar to β-RITs and can be administered in the general hospital. However, if the cytotoxic drugs are resistant to tumor cells, the efficacy is significantly attenuated and this is one of the differences from RIT [57]. A comparative analysis between RIT and other upcoming therapies including ADCs is needed for choosing NHL therapy.

## Single-domain antibodies

The toxicities including myelotoxicity in full mAb-based RIT are related to the long blood residence time of mAb (several days). In addition, its image acquisition is late because of slow blood clearance. The large molecular weight of mAb (around 150 kDa) impairs tissue penetration and binding to hidden antigens. To improve these disadvantages, smaller mAb-derived fragments have been engineered. Single-domain antibodies (sdAbs) are small antigen-binding fragments generated from heavy chain-only antibodies found in Camelidae [58]. In general, sdAbs bind to their targets with high affinity and specificity. Due to their small molecular weight (10–15 kDa), sdAbs show faster blood clearance and better tissue penetration compared with full-size mAbs. Krasniqi et al. [59] assessed the efficacy of radiolabeled anti-CD20 sdAbs using human CD20^+^ lymphoma mouse models. In this investigation, gallium-68 (^68^Ga)-labeled anti-CD20 sdAb was utilized for PET imaging and ^177^Lu-labeled sdAb was administered to treat CD20^+^ lymphoma cells. In the imaging using ^68^Ga-labeled anti-CD20 sdAb, the tumor uptake was specific and the accumulation in nontarget organs (except the kidneys) was low. Although both ^177^Lu-labeled anti-CD20 sdAb and ^177^Lu-labeled rituximab prolonged the median survival rate of treated mice to the same degree, absorbed doses to healthy organs except the kidneys were much higher for ^177^Lu-labeled rituximab in comparison with ^177^Lu-labeled anti-CD20 sdAb. These results indicate that radiolabeled anti-CD20 sdAbs are promising theragnostic approaches to target CD20^+^ NHL.

Collectively, sdAb theoretically shows the high-affinity ability. However, there is no clear evidence of superior specificity of sdAb so far and further studies are required for its clinical application.

### Peptides

Peptide-drug conjugates (PDCs) demonstrate higher cellular permeability and drug selectivity compared with ADCs. The surface expression of C-X-C chemokine receptor type 4 (CXCR4) is up-regulated in some hematological malignancies including NHL [60]. In RIT for NHL, radiolabeled peptides targeting CXCR4 have been studied. The efficacy of ^90^Y-labeled peptide-based ligand (pentixather) along with RIT targeting CD20 or CD66 has been tested in 6 patients with relapsed, refractory DLBCL, which were followed by chemotherapy and allogeneic stem cell transplantation [61]. Of the 4 patients who were available for assessment of responsiveness (one patient died of central nervous system aspergillosis and another died of pneumogenic sepsis), treatment with ^90^Y-labeled pentixather showed a PR in two (both treated with additional RIT targeting CD20 or CD66) and a mixed response in the remaining two.

### PRIT

To improve RIT delivery of therapeutic radionuclides, PRIT was originally proposed by Goodwin et al. [7]. In PRIT, antibody and radionuclide are administered separately. In the first step, the non-radiolabeled antibody is administered and allowed to be maximally accumulated in the tumor sites. In the second step, a secondary radiolabeled molecule with a high affinity for the antibody is injected. PRIT could potentially augment the dose of radionuclides delivered to tumors and improve the tumor-to-normal tissue ratios for RIT.

In streptavidin (SA)-biotin-based PRIT, an SA-conjugated antibody targeting a tumor antigen is administered, and subsequently radiolabeled biotin is injected into the tumor-localized SA. SA-biotin-based PRIT that targets CD20 showed improved biodistributions of radioactivity, reduced toxicity, and markedly increased therapeutic efficacy in a preclinical model of Burkitt lymphoma (Ramos) compared with conventional RIT [62, 63]. Frost et al. [64] compared the efficacy of ^90^Y with that of ^177^Lu for SA-biotin PRIT targeting CD20 in nude mice subcutaneously injected with either Burkitt lymphoma (Ramos) or Granta-519 (mantle cell lymphoma) xenografts. The mean absorbed radiation dose to lymphoma was more than twice as high for ^90^Y as for ^177^Lu. ^90^Y was superior to ^177^Lu for SA-biotin-based PRIT in this study. Pagel et al. [65] compared SA-biotin PRIT targeting CD20, CD22, or human leukocyte antigen (HLA)-DR with conventional RIT in preclinical mouse models using Burkitt lymphoma (Ramos and Raji) or transformed FL (FL-18) xenografts. This investigation showed the marked superiority of SA-biotin PRIT for each of the targets compared with conventional RIT. Park et al. [66] reported ^213^Bi-DOTA-biotin injection following anti-CD20 1F5 tetravalent single-chain variable fragment [(scFv)_4_] SA fusion protein (FP) suppressed lymphoma growth significantly compared with ^213^Bi-DOTA-biotin following non-binding control CC49(scFv)_4_ SA FP in a Ramos xenograft model. ^213^Bi-labeled SA-biotin PRIT targeting CD20 was well tolerated with minimal toxicities and a favorable biodistribution profile. In the phase I/II study, an anti-CD20 antibody (C2B8) conjugated to SA was administered to patients with relapsed NHL [67]. Six of seven patients who were treated with 30 mCi/m^2^ or 50 mCi/m^2^ of ^90^Y-DOTA-biotin achieved objective responses (3 CR and 1 PR). Transient grade III hematological toxicity was presented in five of the seven patients who received 30 mCi/m^2^ or 50 mCi/m^2^ of ^90^Y-DOTA-biotin. The estimate of tumor to whole body dose ratio is high (38:1) in this study. These results indicate the efficacy of PRIT is encouraging and its toxicity is mild. In a subsequent multicenter, phase I study, a novel tetrameric single-chain anti-CD20-SA FP (B9E9FP) followed by ^90^Y-DOTA-biotin was evaluated in 14 patients with relapsed NHL [68]. A high ratio of average tumor to whole-body radiation dose (49:1) was observed with no significant therapy-related hematological toxicity. Treatment with ^90^Y-DOTA-biotin (555 MBq/m^2^) showed only 2 CR and 1 PR, indicating a dose escalation study using ^90^Y-DOTA-biotin is required in future studies. Summary of preclinical and clinical investigations in PRIT of NHL is shown in Table 5 and Table 6.

**Table 5:** Summary of preclinical investigations in PRIT of NHL

Radionucleotide	Lymphoma	Targeting antibody	Comparison with conventional RIT	Reference
^90^Y-DOTA-biotin	Burkitt lymphoma (Ramos)	Anti-CD20 1F5-SA	Yes	[62, 63]
		Anti-CD20 1F5-SA	No	[64]
		Anti-CD20 2H7-Fc-C825 bsMAb	No	[69]
	Burkitt lymphoma (Ramos and Raji), transformed FL (FL-18)	Anti-CD20 1F5-SA, anti-CD22 HD39, anti-HLA-DR Lym-1	Yes	[65]
	Burkitt lymphoma (Namalwa)	Anti-CD38 028-Fc-C825 bsMAb	No	[70]
	Mantle cell lymphoma (Granta-519)	Anti-CD20 1F-5SA	No	[64]
^177^Lu-DOTA-biotin	Burkitt lymphoma (Ramos)	Anti-CD20 1F-5SA	No	[64]
	Mantle cell lymphoma (Granta-519)	Anti-CD20 1F-5SA	No	[64]
^213^Bi-DOTA-biotin	Burkitt lymphoma (Ramos)	Anti-CD20 1F5(scFv)_4_ SA	Yes	[66]
^90^Y-DOTA-histamine-succinyl-glycine (HSG)	Burkitt lymphoma (Ramos)	Anti-CD20 IMMU-106 bispecific mAb (bsMAb)	Yes	[71]
		Anti-CD20 TF4, trivalent-Fab bsMAb	Yes	[72]

**Table 6:** Summary of clinical investigations in PRIT of NHL

Targeting antibody	Main finding	Reference
Anti-CD20 C2B8/SA	Anti-CD20 antibody (C2B8) conjugated to SA followed by 30 mCi/m^2^ or 50 mCi/m^2^ of ^90^Y-DOTA-biotin was administered to patients with relapsed NHL in a phase I/II study. Six of seven patients exhibited objective responses (3 CR and 1 PR). Five of the seven patients showed transient grade III hematological toxicity. The estimate of tumor to whole body dose ratio was 38:1.	[67]
Anti-CD20 B9E9(scFv)_4_ SA	Anti-CD20 B9E9(scFv)_4_ SA followed by ^90^Y-DOTA-biotin was evaluated in patients with relapsed NHL in a phase I study. Three of fourteen patients who received ^90^Y-DOTA-biotin had objective tumor responses (2 CR and 1 PR). No significant hematologic toxicities were reported. The ratio of average tumor to whole-body radiation dose was 49:1.	[68]

Although SA-biotin PRIT targeting CD20 in NHL shows remarkable efficacy in preclinical models, its immunogenicity and the interference of endogenous biotin raise some concerns in clinical application [73]. Sharkey et al. [71] generated a novel bsMAb by coupling the Fab of a humanized anti-CD20 antibody to the Fab of a murine anti- HSG antibody. In this study, nude mice bearing subcutaneous Burkitt lymphoma (Ramos) were treated with the bsMAb, and then, 48 h later, ^90^Y-HSG was administered. The antitumor effects in preclinical models treated with the pre-targeted ^90^Y-HSG peptide were significantly improved compared with the directly radiolabeled ^90^Y-anti-CD20 antibody. The same group subsequently reported that the tumor accretion and retention were improved by the novel dock and lock (DNL) recombinant construct binding divalently to CD20 [72]. Green et al. [69] engineered a bispecific FP composed of an anti-human CD20 antibody (2H7) and an anti-^90^Y-DOTA scFv antibody (C825), which captures ^90^Y-DOTA with very high-affinity. The tumor-to-normal tissue ratios of distribution were superior for the bispecific FP (2H7-Fc-C825) in comparison with SA-biotin. In preclinical models of Burkitt and mantle cell lymphoma, 2H7-Fc-C825 PRIT exhibited higher efficacy and lower myelosuppression than anti-CD20 (1F5)-SA conjugate PRIT. This group also tested a bispecific antibody targeting ^90^Y-DOTA-biotin and CD38. In preclinical models of multiple myeloma and NHL, the CD38-bispecific construct demonstrated excellent target-to-non-target ratios and better survival compared with SA-biotin-based PRIT [70].

### Immuno-PET

Immuno-PET is a radionucleotide imaging combining the sensitivity of PET with the specificity of mAb. Single-site tissue biopsy may not represent the entire burden of the disease because tumor antigen expressions are different from site to site. In contrast, immuno-PET has the potential to accurately evaluate tumor heterogeneity and provide information about therapeutic response. Several lines of preclinical studies have shown that immuno-PET allows non-invasive evaluation of global target levels *in vivo* [74–78].

Intact mAbs including anti-CD20 rituximab have a long biological half-life (several days) due to slow blood clearance. Thus, short half-life radionuclides such as ^18^F (109 min) and ^68^Ga (68 min) are not suitable for this approach in terms of biodistribution in the body. Immuno-PET using cuprum-64 (^64^Cu)-DOTA-rituximab is feasible to evaluate human CD20-expressing lesions in human CD20 transgenic mice because the half-life of ^64^Cu is moderate (12.7 h) [76]. In CD20-positive Raji xenograft models, tumor uptake was specifically detected by immuno-PET with ^64^Cu-DOTA-rituximab [77]. Lee et al. [79] reported lymphoma lesions in 2 NHL patients could be detected more sensitively in immuno-PET using ^64^Cu-DOTA-rituximab compared with ^18^F-FDG PET. ^124^I has a long half-life (4.18 days), but it fails to remain in the cells after internalization and shows unspecific thyroid accumulation.

Among positron-emitting radioisotopes, ^89^Zr is particularly suitable for immuno-PET imaging because ^89^Zr has enough half-life (3.27 days) to analyze the biodistribution of intact mAb. Immuno-PET using ^89^Zr can detect CD20 expressions in a preclinical mouse model [75, 78]. A pilot study using ^89^Zr on 6 patients with relapsed/refractory DLBCL showed there is a correlation between tumor uptake of ^89^Zr-rituximab and CD20 expression [80]. Tumor targeting by ^89^Zr-rituximab is better in the patients without rituximab preloading compared with the rituximab preloading group, suggesting RIT delivery will be affected by preload of non-radiolabeled rituximab [81].

Obinutuzumab binds to CD20 in a different epitope from rituximab. Obinutuzumab is reported to show a slower internalization rate in comparison with rituximab, and immuno-PET using radiolabeled obinutuzumab has the potential to be superior to radiolabeled rituximab immuno-PET [82, 83]. Obinutuzumab-based immuno-PET tracers produced high-contrast images showing CD20 expression in both human CD20^+^ lymphoma xenograft model and human CD20 transgenic mice [83, 84].

CXCR4 can be utilized for tumor detection and assessment of therapeutic response by immuno-PET. In a preclinical Daudi Burkitt lymphoma model, ^64^Cu-labeled, CXCR4-targeting peptide (pentixather) for immuno-PET showed high stability and favorable resolution [85]. In 4 patients including 1 relapsed DLBCL, immuno-PET using ^68^Ga-labeled, CXCR4-targeting peptide (pentixafor) resulted in excellent tumor uptake and contrast [86].

Future development of newer generation antibodies could promote clinical application of immuno-PET. Utilizing sdAb and peptide ligands will improve tumor penetrance and enable earlier imaging of its small size. bsMAb recognizes two different targets and provides better biodistribution *in vivo* compared with conventional antibodies. Identification of novel lymphoma surface makers other than CD20 will provide better imaging options for NHL.

Taken together, immuno-PET helps to evaluate the pharmacokinetics and tumor delivery in RIT. Immuno-PET enables the tracking of cells throughout the body; therefore, the development of immuno-PET can accelerate not only the clinical application of RIT but also other therapies such as chimeric antigen receptor (CAR)-T cell therapy.

## Conclusions

RIT using anti-CD20 mAbs labeled with β-emitting radionucleotides has shown excellent anti-lymphoma effect and reduced toxicity in the therapy of relapsed/refractory indolent NHL or transformed indolent NHL. Previous clinical studies have shown RIT has the potential to be used as not only a first-line therapy but also consolidation and myeloablative conditioning before stem cell transplantation of advanced-stage indolent or aggressive NHL. Although β-particle RITs including ^90^Y-ibritumomab tiuxetan and ^131^I-tositumomab are highly effective for NHL, the number of patients referred for these RITs has gradually decreased in the world over the last two decades due to competing novel therapies and difficulty in performing them in the general hospital. However, targeting surface antigens other than CD20 is also promising and novel therapeutic approaches have continued to be developed in RIT. Recent studies have shown that α-particle RIT is highly effective in preclinical models of NHL. Efficient developments in radiochemistry will reduce production costs and contribute to the increased availability of α-particle RIT. Furthermore, novel treatment strategies such as PRIT have recently made progress and have been expected to be applied clinically to the treatment of NHL. Immuno-PET can provide valuable information about tumor heterogeneity and quantification of antigen expressions as theragnostic approach. The theragnostic approaches including immuno-PET will be vital in monitoring the response of future RIT and other therapies.

Overall, the concept of RIT allows for reduced toxicity and enhances the therapeutic effect of mAbs. RIT is suitable for patients with relapsed or refractory NHL or those who are ineligible for intensive therapies. Further clinical trials will exhibit the potential of future RIT in treating patients with NHL.

## Abbreviations

ADCs: antibody-drug conjugates
bsMAb: bispecific monoclonal antibody
CD20: cluster of differentiation 20
CR: complete response
CRu: complete response unconfirmed
CXCR4: C-X-C chemokine receptor type 4
DLBCL: diffuse large B-cell lymphoma
DOTA: 1,4,7,10-tetraazacyclododecane-1,4,7,10-tetraacetic acid
FDA: Food and Drug Administration
FDG: fluorodeoxyglucose
FL: follicular lymphoma
FP: fusion protein
HSG: histamine-succinyl-glycine
mAb: monoclonal antibody
NHL: non-Hodgkin’s lymphoma
ORR: overall response rate
PET: positron emission tomography
PFS: progression-free survival
PR: partial response
PRIT: pre-targeted radioimmunotherapy
RIT: radioimmunotherapy
SA: streptavidin
sdAbs: single-domain antibodies
(scFv)_4_: tetravalent single-chain variable fragment
TCMC: 1,4,7,10-tetrakis(carbamoylmethyl)-1,4,7,10-tetraazacyclododecane
^131^I: iodine-131
^177^Lu: lutetium-177
^18^F: fluorine-18
^211^At: astatine-211
^212^Pb: plumbum-212
^213^Bi: bismuth-213
^225^Ac: actinium-225
^227^Th: thorium-227
^64^Cu: cuprum-64
^68^Ga: gallium-68
^90^Y: yttrium-90

## Declarations

### Copyright

© The Author(s) 2024.
